# Co-Detection of Virulent *Escherichia coli* Genes in Surface Water Sources

**DOI:** 10.1371/journal.pone.0116808

**Published:** 2015-02-06

**Authors:** Thando Ndlovu, Marcellous Le Roux, Wesaal Khan, Sehaam Khan

**Affiliations:** 1 Department of Biomedical Sciences, Faculty of Health and Wellness Sciences, Cape Peninsula University of Technology, PO Box 1906, Bellville, 7535, South Africa; 2 Department of Microbiology, Faculty of Science, Stellenbosch University, Private Bag X1, Stellenbosch, 7602, South Africa; Wageningen University and Research Centre, NETHERLANDS

## Abstract

McNemar’s test and the Pearson Chi-square were used to assess the co-detection and observed frequency, respectively, for potentially virulent *E. coli* genes in river water. Conventional multiplex Polymerase Chain Reaction (PCR) assays confirmed the presence of the *aggR* gene (69%), *ipaH* gene (23%) and the *stx* gene (15%) carried by Enteroaggregative *E. coli* (EAEC), Enteroinvasive *E. coli* (EIEC) and Enterohermorrhagic *E. coli* (EHEC), respectively, in river water samples collected from the Berg River (Paarl, South Africa). Only the *aggR* gene was present in 23% of samples collected from the Plankenburg River system (Stellenbosch, South Africa). In a comparative study, real-time multiplex PCR assays confirmed the presence of *aggR* (EAEC) in 69%, *stx* (EHEC) in 15%, *ipaH* (EIEC) in 31% and *eae* (EPEC) in 8% of the river water samples collected from the Berg River. In the Plankenburg River, *aggR* (EAEC) was detected in 46% of the samples, while *eae* (EPEC) was present in 15% of the water samples analyzed using real-time multiplex PCR in the Plankenburg River. Pearson Chi-square showed that there was no statistical difference (p > 0.05) between the conventional and real-time multiplex PCRs for the detection of virulent *E. coli* genes in water samples. However, the McNemar’s test showed some variation in the co-detection of virulent *E. coli* genes, for example, there was no statistical difference in the misclassification of the discordant results for *stx* versus *ipaH*, which implies that the *ipaH* gene was frequently detected with the *stx* gene. This study thus highlights the presence of virulent *E. coli* genes in river water and while early detection is crucial, quantitative microbial risk analysis has to be performed to identify and estimate the risk to human health.

## Introduction

In South Africa water abstracted from rivers is used for irrigation and domestic purposes, often without treatment. It has however, been shown that certain surface water sources contain high levels of fecal contamination. *Escherichia coli* found in surface waters, originates mainly from municipal wastewater discharges, septic leachate, agricultural or storm water run-off, wildlife, or non-point sources of human and animal waste [1–3]. Although most strains of the *E*. *coli* group are non-pathogenic members of the normal intestinal flora, certain strains contain virulent genes that may cause various human-related illnesses, such as urinary tract and respiratory infections, diarrhea and pneumonia. Depending on the phenotypic traits and specific virulence factors concerned, intestinal pathogenic strains of *E*. *coli* have been classified into pathotypes, namely, Enteroaggregative *E*. *coli* (EAEC), Enterohermorrhagic *E*. *coli* (EHEC), Enteroinvasive *E*. *coli* (EIEC), Enterotoxigenic *E*. *coli* (ETEC), Enteropathogenic *E*. *coli* (EPEC) and the Diffusely Adherent *E*. *coli* (DAEC) [4–7]. However, lateral gene transfer from pathogenic microorganisms to *E*. *coli* strains may lead to the emergence of new pathogenic strains [5]. They cause different types of diarrheal diseases due to the presence of specific genes associated with pathogenicity, colonization factors and other virulence factors that are generally absent in non-pathogenic strains of this typical indicator organism.

There are numerous assessment methods for the detection and enumeration of organisms such as fecal bacteria and *E*. *coli* in food, water, wastewater effluents and soils. These methods have evolved from the Multiple Tube Fermentation technique (MTF), that is time-consuming, to the Polymerase Chain Reaction (PCR), which is highly sensitive and specific [8]. Conventional multiplex PCR involves the targeting of multiple genes from the same or different organisms by the use of multiple primer sets in a single reaction tube to produce amplicons of different sizes [9, 10]. Lorusso *et al*. [11] developed a multiplex PCR to detect Verocytotoxin-producing *E*. *coli* in raw ground beef and milk, by the detection of the *wzx*, *stx* I and *stx* II genes. The technique was applied to samples inoculated with the *E*. *coli* O26 strain, which is positive for the named genes, and also for un-inoculated samples where it was shown to be highly sensitive. The authors concluded that the developed multiplex PCR could be further applied to clinical and environmental samples for the detection of the same *E*. *coli* strain, as the major pollutants were successfully identified. In addition real-time PCR is a reliable technique for the identification and measurement of amplicons generated during each cycle of the PCR process, which directly corresponds to the starting concentration of the template [12, 13]. Reischl *et al*. [14] developed a real-time PCR for the identification of the heat labile enterotoxin and the heat stable enterotoxin from ETEC of human origin using a Roche light cycler. They used gene specific primers and hybridization probes during the reaction and they found the assays to be 100% sensitive and specific for both genes. In a previous study conducted by Sidhu *et al*. [15], virulent *E*. *coli* genes were detected, using the real-time PCR, in surface water sources used for potable, non-potable and recreational purposes in Brisbane, Australia during wet and dry periods. Sewage from the surrounding urban communities was thought to be the main source of contamination of the river and dam water. They statistically analyzed the frequency distribution of the virulent *E*. *coli* genes using analysis of variance (ANOVA) between the dry and wet periods, and concluded that the detection of the virulent genes was more prevalent during the wet period than during the dry periods. Results from the study also showed that the EAEC, EPEC and EIEC were more frequently detected, in comparison to the detection of the EHEC strain in surface water sources.

The current study was thus aimed at (i) comparing the applicability and sensitivity of conventional- and real-time multiplex PCRs for the detection of *aggR*, *stx*, *IpaH* and *eae* genes associated with EAEC, EHEC, EIEC and EPEC strains in river water samples, (ii) using the Pearson Chi-square to compare the observed frequency of potentially virulent *E*. *coli* genes across the Berg and Plankenburg River systems by the conventional and real-time multiplex PCRs and (iii) using McNemar’s test to assess the co-detection of virulent *E*. *coli* genes in river water samples.

## Materials and Methods

### 2.1 Collection of Surface Water Samples

Sampling was conducted for a period of six months [samples were collected every two weeks (weeks 1 to 28)] during dry periods (no rainfall 48 hours prior to sampling) and wet periods (experienced in weeks 9 and 13 with rainfall recorded less than 10 hours prior to sampling). A five liter water sample was collected by immersing a sterile Nalgene bottle into the river at the point closest to the informal settlements of Kayamandi and Mbekweni, situated along the Plankenburg- and Berg Rivers in the Western Cape, South Africa, respectively, and was transported to the laboratory on ice to maintain a temperature below 4°C. A total of 13 water samples were collected from each respective river system (n = 26) and were processed within six hours of sampling. These sites were selected as significantly high fecal coliform and *E*. *coli* counts (p < 0.05) of up to 3.5 x 10^7^ and 1.7 x 10^7^microorganisms/100 ml, respectively, for both river systems were obtained throughout the sampling period in the studies conducted by Paulse *et al*. [16, 17]. Permission was not required from any authority or body to collect water at any of these locations. No endangered or protected species were involved during the collection of the water (Plankenburg River GPS coordinates: 33°55&rsquo;36.7"S, 18°51&rsquo;05.8"E; Berg River GPS coordinates: 33°40&rsquo;14.4"S 18°59&rsquo;05.5"E).

**2.1.1 Enumeration of Fecal Coliforms in Surface Water Samples.** The multiple tube fermentation technique as previously modified by Barnes [1] and employed by Paulse *et al*. [16, 17], was used to enumerate fecal coliforms.

### 2.2 Culturing of Control Microorganisms and DNA Extraction

*Escherichia coli* strains that were used as positive controls in the PCRs were obtained from the National Institute for Communicable Diseases (Johannesburg, SA). Strain O157:H7 was used as a positive control for EHEC, B170 for EPEC, 3591–87 for EAEC and ATCC 43892 for EIEC. The extraction and purification of DNA from the control strains was performed using the boiling method adapted from Watterworth *et al*. [18] and the High Pure PCR Template Preparation Kit (Roche Diagnostics, Germany) according to the manufacturer’s instructions. For the boiling method, pure cultures of the microorganisms were grown on nutrient agar (NA) at 37C for 18–24 hours, and a single colony was inoculated into Luria Bertani (LB) broth and incubated for 18–24 hours at 37C. One milliliter of broth aliquot was centrifuged at 14 000 rpm for 10 minutes, the supernatant was discarded and the pellet was resuspended in 100 μl of sterile double distilled water and boiled in a 95C water bath for 15 minutes. The suspension was then cooled on ice for 10 minutes, and centrifuged at 14 000 rpm for 5 minutes with the supernatant (DNA was contained in the supernatant) transferred into a sterile 1.5 ml eppendorf tube.

**2.2.1 Extraction of Bacterial DNA from Surface Water Samples.** Deoxyribonucleic acid extraction from surface water samples was performed within 24 hours from collection. Microbial cells were harvested by centrifuging 500 ml of surface water at 7 000 rpm for 20 minutes. The pellet was then incubated in 2 ml of LB broth for 6 hours at 37C, after which the extraction and purification of DNA was performed using the boiling method adapted from Watterworth *et al*. [18]. The protocol was followed as described in section 2.2.

**2.2.2 Conventional Multiplex PCR for the Detection of Virulent *E*. *coli* genes in Surface Water Samples.** The primers used to amplify each target gene using conventional multiplex PCR assays are indicated in Table 1. The primers used in the study were selected based on their sensitivity and specificity shown in multiplex PCR assays from previous studies [19–24] targeting *E*. *coli* strains applied on various samples. However, since the primer set used for EIEC, targeting the *ipaH* gene, amplifies a product for both EIEC and *Shigella* sp., singleplex PCR with primers that were *Shigella* and *E*. *coli* specific were also used (data not shown). The amplified products were confirmed by sequencing. The results in the paper thus represent the EIEC positive and *Shigella* negative samples. The PCR reaction mixture comprised of 1X PCR reaction buffer, 10 mM Tris-HCl (pH 8.3), 50 mM KCl, 0.1% Triton X-100, 2.5 mM MgCl_2_, 0.25 mM dNTP mix, 0.1 μM IpaH1 and IpaH2 primers (*ipaH* gene), 0.125 μM of SK1 and SK2 primers (*eae* gene), 0.25 μM VTcom-u, VTcom-d primers (*stx* gene), 0.2 μM AggRks1 and AggRkas2 primers (*aggR* gene), 5 U of Go Taq DNA polymerase, 10 μl of template DNA and double sterile distilled water to adjust the final volume to 60 μl. A positive control containing known EAEC, EHEC, EIEC and EPEC template DNA samples was always included. The PCR reaction program was performed using the My cycler thermal cycler (Biorad, USA). The cycling program was performed using the initial template denaturation step at 95C for 2 minutes followed by 30 cycles of amplification (denaturing at 95C for 1 minute, annealing at 48C for 1 minute, extension at 72C for 1 minute) and the final extension at 72C for 7 minutes. The amplified PCR products were purified using the High Pure PCR Product Purification kit (Roche Diagnostics, Germany) or Wizard SV Gel and PCR clean up system (Promega, USA) and sequenced in accordance with the BigDye Terminator Version 3.1 Sequencing Kit. All the sequences were verified using the online Basic Local Alignment Search Tool (BLAST) program obtained from the National Centre for Biotechnology Information website [25].

**Table 1:** Primer sequence and predicted sizes of amplicons in conventional and real-time multiplex PCRs.

Organism	Primer name and Sequence (5′-3′)	Target gene	Product size (bp)	References
EAEC	AggRks1- GTATACACAAAAGAAGGAAGC	aggR	254	[40]
	AggRkas2- ACAGAATCGTCAGCATCAGC			
EHEC	VTcom-u- GAGCGAAATAATTTATATGTG	stx	518	[41]
	VTcom-d- TGATGATGGCAATTCAGTAT			
EIEC	IpaH1- GTTCCTTGACCGCCTTTCCGATACCGTC	ipaH	619	[42]
	IpaH2- GCCGGTCAGCCACCCTCTGAGAGTAC			
EPEC	SK1- CCCGAATTCGGCACAAGCATAAGC	eae	881	[43]
	SK2- CCCGGATCCGTCTCGCCAGTATTCG			

**2.2.3 Real-time Multiplex PCR for the Detection of Virulent *Escherichia coli* genes in Surface Water Samples.** The real-time multiplex PCR protocol was carried out with the 0.75X Sybr green master mix (Qiagen, USA), 0.5 μM of SK1 and SK2 primers (*eae* gene), 0.125 μM IpaH1 and IpaH2 primers (*ipaH* gene), 0.25 μM VTcom-u, VTcom-d primers (*stx* gene), 0.25 μM AggRks1 and AggRkas2 primers (*aggR* gene), 10 μl of template DNA and double distilled water was used to adjust the total reaction volume to 40 μl. No additives (Tris-HCl and Triton X-100) were included in the real-time multiplex PCR as they were found to inhibit amplification of the virulent *E*. *coli* genes in control strains. A positive control containing known EAEC, EHEC, EIEC and EPEC template DNA samples was always included. The real-time multiplex PCR conditions used for river water sample analysis, involved an initial enzyme activation and denaturation step at 98C for 3 minutes, followed by 35 cycles of amplification (98C for 5 seconds, 48C for 5 seconds and 72C for 30 seconds) and a final extension at 72C for 2 minutes. Amplifications for each gene in each microorganism were performed using the MJ MiniOpticon real-time thermal cycler (Bio-Rad, USA).

### 2.3 Statistical Analysis

The results obtained for the virulent *E*. *coli* analysis of the water samples collected from the Berg- and Plankenburg River systems was assessed using the statistical software package Statistica Ver 11.0 174 (Stat Soft Inc, Tulsa, USA). The Pearson Chi-square was used to compare the observed frequency of virulent *E*. *coli* genes across the Berg and Plankenburg River systems using the conventional and real-time multiplex PCRs. The gene specific PCR assays representing each of the virulent *E*. *coli* genes were analyzed using McNemar’s test in order to determine the relationship between or co-detection of each virulent gene in river water samples. The data obtained from the conventional and real-time multiplex PCR assays were firstly assigned values to represent their presence or absence in tested water samples, a positive PCR product was assigned the value 1 (present), and when no PCR product was observed, it was assigned the value 0 (absent). In all hypothesis tests, a significant level of 5% was used as standard.

## Results

### 3.1 Enumeration of Fecal Coliforms in Surface Water Samples

The fecal coliform counts in water samples collected from the Berg River ranged from 1.1 × 10^3^ microorganisms/100 ml in week 1 to 1.4 × 10^4^ microorganisms/100 ml in week 28. The highest fecal coliform count of 1.4 × 10^6^ microorganisms/100 ml was obtained in weeks 19 and 21, while the lowest count of 1.1 × 10^3^ microorganisms/100 ml was obtained in week 1.

In addition, the fecal coliform counts for the Plankenburg River system ranged from 1.1 × 10^3^ microorganisms/100 ml in week 1 to 9.2 × 10^6^ microorganisms/100 ml in week 28. The highest fecal coliform count of 9.2 × 10^6^ microorganisms/100 ml was obtained in weeks 21 and 28, while the lowest count of 1.1 × 10^3^ microorganisms/100 ml was obtained in week 1.

### 3.2 Comparison of results obtained by the conventional and real-time PCRs for the detection of virulent *Escherichia coli* genes

**Fig 1:**
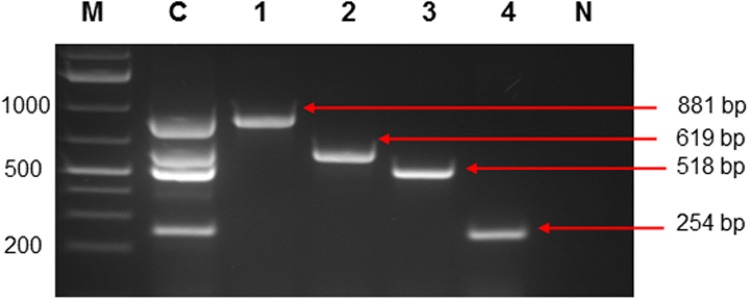
Control strains of *E*. *coli* using virulent specific primers. Lane M: Generuler 1 kb plus (Fermentas); lane C: Multiplex of e*ae* (EPEC), *ipaH* (EIEC), *stx* (EHEC), *aggR* (EAEC); lane 1: *eae* (881 bp); lane 2: *ipaH* (619 bp); lane 3: *stx* (518 bp); lane 4: *aggR* (254 bp) and lane N: negative control.

A multiplex PCR performed on control *E*. *coli* strains illustrating the multiplex PCR amplicons (lane C) and single reaction amplicons for *eae* (lane 1), *ipaH* (lane 2), *stx* (lane 3) and *aggR* (lane 4) is shown in Fig. 1. A Pearson Chi-square test was used for the comparison of results obtained by the conventional and real-time multiplex PCRs for the detection of virulent *E*. *coli* genes throughout the sampling period (Table 2). Conventional multiplex PCR assays were applied to water samples collected from the Berg and Plankenburg River systems for the detection of the EHEC (*stx* gene), EIEC (*ipaH* gene), EPEC (*eae* gene) and EAEC (*aggR* gene) strains. The *eae* gene was not detected throughout the sampling period by the conventional multiplex PCR technique in 26 water samples collected in total (13 samples collected per site) from the Berg and Plankenburg River systems. The *aggR* gene carried by the EAEC strain was the single most prevalent gene (69%) detected in the Berg River samples, while in the Plankenburg River, it was detected in 23% of the samples. The *ipaH* and *stx* genes were not detected in the Plankenburg River samples, but were detected in 23% and 15%, respectively, of the Berg River water samples (Table 2).

**Table 2:** Pearson Chi-square observed frequency for *E*. *coli* virulent genes in the Berg and Plankenburg River systems using the Conventional- and Real-time PCR assays.

Site	Technique	% Detection of *Escherichia coli* virulent genes			
		*eae* (EPEC)	*ipaH* (EIEC)	*stx* (EHEC)	*aggR* (EAEC)
Berg River	*P values*	*p = 0.23*	*p = 0.66*	*p = 1.00*	*p = 1.00*
	Conventional PCR	0% (0/13)	23% (3/13)	15% (2/13)	69% (9/13)
	Real-time PCR	8% (1/13)	31% (4/13)	15% (2/13)	69% (9/13)
Plankenburg River	*P values*	*p = 0.086*	*n/a*	*n/a*	*p = 0.21*
	Conventional PCR	0% (0/13)	0% (0/13)	0% (0/13)	23% (3/13)
	Real-time PCR	15% (2/13)	0% (0/13)	0% (0/13)	46% (6/13)

Real-time multiplex PCR assay using the Sybr green master mix (Qiagen, Netherlands) enabled the detection of the virulent *E*. *coli* genes in contaminated surface water. The Pearson Chi-square frequency for the detection of the virulent genes in each river system using real-time multiplex PCR is depicted in Table 2. For the 13 water samples collected from the Berg River the *aggR*, *ipaH*, *stx* and *eae* genes were present in 69%, 31%, 15% and 8% of the samples collected, respectively. In the Plankenburg River system, the *aggR* and *eae* genes were detected in 46% and 15% of the samples, respectively. The *stx* and *ipaH* genes carried by the EHEC and EIEC strains, respectively, were not detected by the real-time multiplex PCR in the Plankenburg River water samples (Table 2). The *aggR* gene was the most prevalent in the Berg River (69%), and in the Plankenburg River (46%) water samples. In contrast, the *eae* gene was only detected in 8% and 15% of the Berg and Plankenburg River samples, respectively, while the *stx* gene carried by the EHEC was not detected in the Plankenburg River system using real-time multiplex PCR during the entire sampling period.

### 3.3 Statistical Analysis of Conventional and Real-time Multiplex PCRs for possible indicator gene

A Pearson Chi-square test was used for the comparison of results obtained by the conventional and real-time multiplex PCRs for the detection of virulent *E*. *coli* genes throughout the sampling period (Table 2). The Pearson Chi-square test showed that there was no significant difference (p > 0.05) between the ability of the two techniques to identify the respective virulent genes (Table 2). The McNemar’s test was then used to determine if one particular virulent gene could be used as a possible indicator for the presence of other virulent genes. It is a test where 2 x 2 classification tables (representative shown in Table 3) with matched pairs of non-parametric data are utilized. In this study it was used to tabulate the results for virulent *E*. *coli* genes obtained from 26 river water samples (on paired dichotomous observations), to test the significance of the difference between detection of the virulent genes [26], using the real-time multiplex PCR. The McNemar’s test could not be performed on results obtained by the conventional multiplex PCR for the Berg and Plankenburg River systems, as there was a low detection of the virulent *E*. *coli* genes. Discordant results, represent pairs of data with a difference (if one gene is present and the other gene is absent in the water sample) and the concordant cells represent pairs of data with no difference (the two genes are either both present or absent in a water sample). The discordant cell results are then used in McNemar’s test to assess the co-detection of virulent genes in water samples. If the McNemar test is significant in misclassification of the virulent genes, it implies that the real-time multiplex PCR has misclassified the co-detection of two genes; therefore one gene could not be used to detect the presence of the other. If the misclassification is not significant (p > 0.05), then it signifies that the real-time multiplex PCR has classified the co-detection of two genes in the same water sample, therefore they are likely to be found in the same water sample.

**Table 3:** Comparison for the detection of ***eae*** and ***aggR*** genes in the Berg and Plankenburg River systems using the real-time multiplex PCR.

EAEC(*aggR*)	EPEC(*eae*)Absent	EPEC(*eae*)Present	Row Totals
Absent	11	0	11
Present	12	3	15
Totals	23	3	26

*P value = 0*.*0015*.

The proportion of water samples classified as positive for *aggR* and *eae* genes was 12% (three water samples), and both virulent genes showed insufficient detection limits in 11 water samples (Table 3). Results for both *aggR* and *eae* genes were discordant in 12 water samples (46%), as all water samples were positive for the *aggR* gene and negative for the *eae* gene. There was a statistical difference (p = 0.0015) in the McNemar’s test for the misclassification of the *aggR* and *eae* genes in water samples, which implies that the *aggR* gene could not indicate the presence of the *eae* gene. There was also a statistical difference (p = 0.0026) in the McNemar’s test for the misclassification of the *ipaH* gene versus *aggR* gene in water samples, which means that the *aggR* gene was not detected in the same water sample with the *ipaH* gene. In addition, a statistical difference (p = 0.0087) in the misclassification of the discordant results for the *aggR* and *stx* genes was recorded, meaning that the *aggR* gene could not indicate the presence of the *stx* gene in the same water sample using the real-time multiplex PCR. In contrast, no statistical difference (p = 0.48) in the misclassification of the discordant results for *ipaH* versus *stx* genes in water samples was recorded, which implies that these virulent genes could occur in the same water sample. In addition, no statistical difference (p = 1.0) in the discordant results for the misclassification of the *stx* versus *eae* and *eae* versus *ipaH* genes in river water samples was recorded, meaning that there was an equal chance of detecting the virulent genes together in the same sample.

## Discussion

Fecal coliforms, which includes the *E*. *coli* group, generally serve as an indication of the level of sewage or fecal contamination, from warm-blooded animals (including humans), in a water source. Significantly high (p < 0.05) fecal coliform counts were then obtained for both river systems throughout the sampling period. These results correlate with the fecal coliform counts observed in previous studies conducted, where water samples collected from the Berg and Plankenburg River systems were also analyzed for the fecal indicator groups [1, 16, 17, 27]. In the Berg and Plankenburg River systems, all fecal coliform counts obtained exceeded the stipulated guidelines of 0 and 2000 microorganisms/100 ml stipulated by DWAF [28] for water used in domestic and recreational purposes, respectively. In addition, the significantly high (p < 0.05) fecal coliform counts obtained in sampling weeks 19, 21 and 28, could be ascribed to the fact that these sampling sessions were conducted in the summer period, where the ambient and water temperatures had started to increase significantly (> 25°C) [29].

During the entire study period, the conventional and real-time multiplex PCRs were compared for their applicability in the detection of virulent *E*. *coli* genes in river water samples. The DNA extracted from river water samples was then screened for the presence of *aggR*, *stx*, *ipaH* and *eae* virulent genes usually carried by EAEC, EHEC, EIEC and EPEC strains, respectively. The most prevalent virulent gene (Table 2) detected in the Berg River and Plankenburg River systems using both techniques was the *aggR* gene found in the EAEC strain. The results obtained in this study were thus in agreement with previous studies performed in Tunisia and in the Province of Gauteng, South Africa [19; 30] where the *aggR* gene was detected more frequently in comparison to other virulent genes. Salem *et al*. [19] detected the *aggR* gene carried by the EAEC strain in 16.6% of the wastewater influent and only 26.6% was removed by the treatment processes in the wastewater treatment plants in central Tunisia. A study performed by Omar and Barnard [30], also detected the *aggR* gene associated with EAEC in all untreated sewage samples and in approximately 57% of treated sewage effluent samples ready to be released into the environment.

Real-time PCR detected the *ipaH* gene associated with the EIEC in 31% (no statistical difference, p = 0.66) of the water samples collected in the Berg River. The presence of the *ipaH* gene in EIEC could also indicate the presence of *Shigella dysentery*, since the gene is found in more than one pathotype [31]. However, as indicated in Section 2.2.2 singleplex PCR with primers that were specific for *Shigella* and *E*. *coli*, respectively, were also utilized (data not shown). Only samples that were EIEC positive and *Shigella* negative were included in the current study. In a previous study, performed on environmental water samples, singleplex PCR enabled the identification of the *ipaH* gene specific for the EIEC, which was present in 11 water samples [32]. Enteroinvasive *E*. *coli* have also been implicated in food-borne outbreaks as the causative agent of diarrhea amongst travelers.

Overall, the occurrence of the EPEC strain was much lower than the occurrence of the EAEC strain in the two river systems throughout the sampling period. The *eae* gene which codes for the intimin protein, had a low detection rate in the Berg (8%) and Plankenburg (15%) River systems using the real-time multiplex PCR and was not detected using conventional multiplex PCR. This gene is required for intimate attachment to host epithelial cells in both the EHEC and EPEC strains, but the primer set used in this study was specific for the *eae* gene found in the EPEC strain, which was confirmed by sequencing and using the online BLAST program. Previous studies also detected the *eae* gene using singleplex and conventional multiplex PCR in sewage polluted seawater samples in Hong Kong and the effluent of a wastewater treatment plant in the Gauteng Province of South Africa, respectively, [30, 33]. A study conducted in Shongwe hospital (Mpumalanga province, South Africa), between February 1985 and January 1986 on pediatric patients with diarrhea, revealed that 27.6% of cases were caused by the EPEC strain [34].

In the current study, the *stx* gene (EHEC) was detected in 15% of the water samples processed using the conventional and real-time multiplex PCR from the Berg River system and was not detected in the Plankenburg River. A study conducted by Doughari *et al*. [35] confirmed the presence of the verotoxins 1 and 2, which is also associated with the EHEC strain in the Berg River system using the antibody-based rapid slide agglutination assay with a Duopath Kit (Merck, Johannesburg, South Africa). The primer sets used in this study targeted a region common for both the *stx1* and *stx2* genes found in the EHEC strain, which causes hemolytic uremic syndrome and hemorrhagic colitis in humans [36]. The detection of the *stx* gene in river water is of concern, as this water is used for various purposes without treatment, and humans can easily be infected with this microorganism.

In 2011, an outbreak of diarrhea and hemolytic-uremic syndrome caused by a rare strain of shiga-toxin producing *E*. *coli* (STEC) was reported in Germany [37]. The strain was said to be more virulent compared to most shiga-toxin producing *E*. *coli*. Prior to this outbreak, the first reported incident of human STEC O104:H4 infections occurred in 2001 in Cologne, Germany. Between 2001 and 2011 only sporadic outbreaks of human STEC O104:H4 were recorded. Comparative genomic studies showed that the virulence genes of STEC O104:H4 strains isolated from sporadic cases and outbreaks in 2001 until 2011 had fundamental differences to other STEC and EHEC strains. The STEC O104:H4 isolates were shown to share virulence properties with enteroaggregative *E*. *coli* (EAEC), and all STEC O104:H4 strains isolated for the time period were found to produce Stx2a; a toxin type which is associated with severe clinical outcome in infected patients [38, 39]. This study thus showed that the *stx* gene could not be used as a pure indicator for EHEC within Europe and Asia. No reported incidence of STEC O104:H4 has however, been shown to occur within South Africa.

The data obtained for the conventional and real-time multiplex PCR assays was then analyzed using the Pearson Chi-square test to compare the ability of the two techniques to detect virulent genes in river water samples. As indicated, the real-time multiplex PCR procedure was more effective in the frequent detection of virulent *E*. *coli* genes during the entire sampling period, but it was statistically comparable to conventional multiplex PCR as shown by the Pearson Chi-square analysis (Table 2, p values ranging from 0.086 to 1.0). In total, for both river systems the conventional multiplex PCR detected the *aggR* gene in 46% of the 26 river water samples tested, while real-time multiplex PCR detected the same gene in 58% of the 26 river water samples analyzed. The *eae* gene was not detected by conventional multiplex PCR in the Berg and Plankenburg River systems, while real-time multiplex PCR detected the *eae* gene in 12% of the 26 river water samples tested. The *stx* and *ipaH* genes were not detected by either technique in the Plankenburg River system. In contrast, a higher frequency of detection for the *stx* and *ipaH* genes was obtained for the Berg River system using both conventional and real-time multiplex PCR.

McNemar’s test was used to assess the co-detection of virulent genes in water samples collected from the Berg and Plankenburg River systems using the real-time multiplex PCR. Analysis of the real-time multiplex PCR revealed the co-detection of certain virulent genes (*stx* and *ipaH*, *eae* and *ipaH*, *stx* and *eae*) in a surface water source, which implies that more than one pathotype of the *E*. *coli* strain, was present in water. However, the presence of more than one *E*. *coli* pathotype in surface water raises health risks associated with the possibility of severe diarrhea in humans [33], if the water is utilized for domestic or recreational purposes without treatment. In addition, various pathogenic *E*. *coli* strains cause several types of human diarrhea, which implies that exposure to water samples with more than one *E*. *coli* pathotype could lead to severe human illness [6, 33]. While the results of this study highlights the possibility of co-detecting the *ipaH* gene with either the *stx* or *eae* genes and the *stx* with the *eae* gene in a water sample, there is no certainty at this point that one virulent gene can be used as a final indicator for the presence of another virulent *E*. *coli* gene and further studies need to be conducted. In addition, the study reinforces the importance of managing point discharges of pollutants into environmental waters, which are widely used for domestic, irrigational and recreational purposes. Moreover, a better understanding of the prevalence of virulent *E*. *coli* genes in river water could be an important tool in the development of public health risk mitigation strategies.
